# Prostate cancer prognosis after initiation of androgen deprivation therapy among statin users. A population-based cohort study

**DOI:** 10.1038/s41391-021-00351-2

**Published:** 2021-03-31

**Authors:** A. I. Peltomaa, P. Raittinen, K. Talala, K. Taari, T. L. J. Tammela, A. Auvinen, T. J. Murtola

**Affiliations:** 1 University of Tampere, School of Medicine, Tampere, Finland; 2 Department of Mathematics and Systems Analysis, Aalto University, School of Science, Espoo, Finland; 3 Finnish Cancer Registry, Helsinki, Finland; 4 Department of Urology, University of Helsinki and Helsinki University Hospital, Helsinki, Finland; 5 Department of Urology, Tampere University Hospital, Tampere, Finland; 6 University of Tampere, School of Health Sciences, Tampere, Finland

**Keywords:** Cancer epidemiology, Prostate cancer

## Abstract

**Purpose:**

Statins’ cholesterol-lowering efficacy is well-known. Recent epidemiological studies have found that inhibition of cholesterol synthesis may have beneficial effects on prostate cancer (PCa) patients, especially patients treated with androgen deprivation therapy (ADT). We evaluated statins’ effect on prostate cancer prognosis among patients treated with ADT.

**Materials and methods:**

Our study population consisted of 8253 PCa patients detected among the study population of the Finnish randomized study of screening for prostate cancer. These were limited to 4428 men who initiated ADT during the follow-up. Cox proportional regression model adjusted for tumor clinical characteristics and comorbidities was used to estimate hazard ratios for risk of PSA relapse after ADT initiation and prostate cancer death.

**Results:**

During the median follow-up of 6.3 years after the ADT initiation, there were 834 PCa deaths and 1565 PSA relapses in a study cohort. Statin use after ADT was associated with a decreased risk of PSA relapse (HR 0.73, 95% CI 0.65–0.82) and prostate cancer death (HR 0.82; 95% CI 0.69–0.96). In contrast, statin use defined with a one-year lag (HR 0.89, 95% CI 0.76–1.04), statin use before ADT initiation (HR 1.12, 95% CI 0.96–1.31), and use in the first year on ADT (HR 1.02, 95% CI 0.85–1.24) were not associated with prostate cancer death, without dose dependency.

**Conclusion:**

Statin use after initiation of ADT, but not before, was associated with improved prostate cancer prognosis.

## Introduction

Statins reduce blood cholesterol levels, which play a central role in androgen biosynthesis. Statins also limit cancer cell growth through the inhibition of the mevalonate pathway [1] and may inhibit lipogenesis which is important for cancer cells [2]. Furthermore, inhibition of acetyl-CoA modifies immune response. The immune response against tumors provides another possible mechanism for statins’ anti-cancer effects [3]. Recent studies have concentrated on evaluating statins’ efficacy in the treatment of cancer patients [1, 4–7].

In prostate cancer, statin use has been associated with a longer time to disease progression after the primary therapy [1, 4, 5] and reduced disease-specific mortality [6]. Intraprostatic cholesterol metabolism and its upregulation have a key role in the development of castration-resistance during androgen deprivation therapy (ADT) [7]. Concordantly, the survival benefit associated with statin use may be strongest among men treated with ADT. However, there are only a few studies assessing statins’ effect specifically in relation to ADT. Therefore, we evaluated the risk of prostate cancer death after ADT initiation with a special focus on the timing of statin use in relation to ADT.

## Methods

### Study cohort

**Fig. 1:**
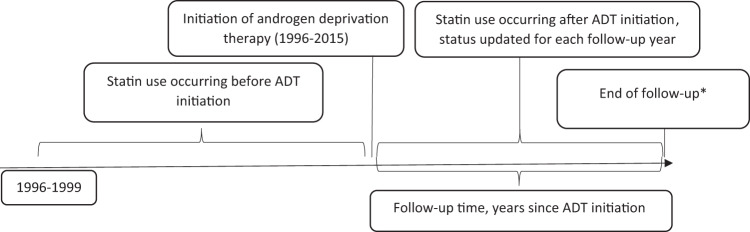
Longitudinal design of the study. Initiation of androgen deprivation therapy (ADT) was defined to calculate statin use occuring before and after ADT. *Death/PSA relapse, emigration, or common closing date 1 January 2016.

Finnish randomized study of screening for prostate cancer [8] is a randomized population-based trial assessing effects of systematic screening with prostate-specific antigen (PSA) on prostate cancer mortality. At baseline, 80 458 men aged 55–67 years residing in the metropolitan areas of Helsinki or Tampere were randomized in 1996–1999 either to be invited for PSA-screening every 4 years or to the control arm without any intervention. The study population was identified from the population register center and linked to the comprehensive Finnish cancer registry (FCR) to exclude all prevalent prostate cancer cases. Incident PCa cases after baseline were identified from patient files and FCR. During 1996–2015 a total of 8253 men in the study population were diagnosed with prostate cancer, with 4428 initiating ADT after diagnosis. These men formed the study cohort for the present study. The longitudinal study design is graphically formatted in Fig. 1.

Information on TNM stage, PSA values (all measurements during 1996–2016), and Gleason score at diagnosis and possible radiation therapy (as primary or adjuvant/salvage treatment) were retrieved from hospital records and laboratory data of Fimlab or HUSLAB, main laboratory services providers in Tampere and Helsinki areas. By combining these tumor characteristics, we were able to categorize patients into prognostic risk groups for biochemical recurrence as defined by European Urological Association [9]. PCa patients with PSA < 10 at diagnosis, localized disease, T1-2a, and Gleason score 6 or lower were classified as low-risk cases. Information on BMI was available for 805 men at the time of screening. PCa cases with PSA above 20, Gleason score above 7, locally advanced disease, T2c-4 or N+ were classified as high-risk cancers. All de novo metastatic (M1) cases were also categorized as high risk. All other PCa cases were grouped into intermediate-risk groups. PSA relapse was defined as two consecutive rises of at least 50% from nadir PSA provided that the final PSA was over 2.

Statistics Finland registers all deaths occurring in Finland. In this analysis deaths with prostate cancer (ICD-10 code C61) as the primary cause of death were defined as prostate cancer deaths. Statistics Finland has also approved (TK-53-1330-18) our research permission.

### Information on medication use

The study cohort was linked to the National prescription database maintained by the social insurance institute (SII) of Finland. SII provides reimbursements for purchases of physician-prescribed medication in Finland. The Finnish reimbursement system has been described in detail previously [10]. ADT use was defined as any purchase of GnRH agonists, GnRH antagonists, or antiandrogens during 1995–2015, as identified by drug-specific ATC codes. Similarly, information on all statins, as well as, antidiabetic drugs, antihypertensive drugs, and non-steroidal anti-inflammatory drugs were obtained.

To complement our data on ADT, information on surgical bilateral orchiectomies performed during 1995–2015 were obtained from the care register for health care (HILMO) maintained by the National Institute for Health and Welfare. Orchiectomies were identified using the nordic classification of procedures code KFC10.

The initiation of ADT use was defined as the date of surgical orchiectomy or the first reimbursement for prescription of any ADT medication, whichever occurred first.

The annual cumulative mg amount of use for each statin during follow-up was calculated by adding together all purchases within a given calendar year. Amount of use was standardized between different statins by dividing the annual total mg amount of purchases with the amount corresponding to a defined daily dose (DDD) as listed by the World Health Organization [11]. Each year with any recorded statin purchases was considered as a year of usage. Cumulative statin DDDs and years of usage were calculated by adding together yearly purchases. Average yearly DDDs, i.e., the intensity of statin use was calculated by dividing yearly cumulative DDDs with a cumulative number of years of usage.

### Statistical analysis

Cox proportional hazards model was used to estimate hazard ratios (HRs) and 95% confidence intervals (95% CIs) for risk of PSA relapse, prostate cancer death, and death due to any cause after initiation of ADT. Follow-up time for these analyses started at ADT initiation and ended in PSA relapse or death, emigration, or common closing date 1 January 2016, whichever came first. We used model adjustment for randomization group, age, tumor risk group, simultaneous use of NSAIDs, antidiabetics, or antihypertensive drugs, and whether the participant received radiation therapy in addition to ADT.

Statin use after ADT initiation was analyzed as a time-dependent variable; status of usage, as well as the cumulative amount, duration, and intensity of use, were updated separately for each follow-up year based on recorded statin purchases. Statin use before ADT initiation was analyzed as the time-fixed variable. Usage status of all other medications (NSAIDs, antidiabetics, and antihypertensive drugs) was not allowed to change on a yearly basis, and participants were regarded as a user if a person had one reimbursed purchase during the follow-up.

To estimate the latency of the risk associations with statin use we performed lag time analyses using statin exposure occurring 1–3 years earlier instead of the contemporary exposure, e.g., for outcomes occurring on the fifth follow-up year we used statin use from the fourth year of follow-up as the exposure in the 1-year lag time analysis.

## Results

### Population characteristics

Of the 4428 ADT-treated patients, 2544 (47.9%) had used statins during the follow-up. During the median follow-up of 6.3 years from the initiation of ADT, there were 482 and 352 deaths due to prostate cancer and 723 and 842 PSA relapses among nonusers and statin users, respectively (Table 1). ADT method among statin users was slightly more often GnRH agonists/antagonists and less often orchiectomy.

**Table 1:** Population characteristics, the cohort of prostate cancer patients treated with androgen deprivation therapy.

	Participants starting ADT	
	Statin use during the follow-up (1996–2015)	
	None	Any
No of men	1884	2544
No of PCa deaths	482 (25.6%)	352 (13.8%)
No of overall deaths	1009 (53.6%)	918 (36.1%)
Median (IQR) follow-up time (years) after ADT initiation	5.5 (2.6–9.4)	6.9 (3.6–10.6)
Mean age at PCa diagnosis (years)	69.0	69.7
Mean age at ADT initiation	70.1	70.9
BMI; median (IQR)	26.0 (23.7–28.7)	26.8 (24.7–29.1)
Tumor stage at diagnosis		
T1–2	1 146 (60.8%)	1 781 (70.0%)
T3–4	737 (39.2%)	763 (30.0%)
Unknown	1 (0.02%)	0
Tumor Gleason grade		
6 or lower	583 (30.9%)	886 (34.8%)
7	671 (35.6%)	957 (37.6%)
8–10	575 (30.5%)	660 (25.9%)
Metastatic disease at diagnosis (M1)	316 (16.8%)	240 (9.4%)
PSA level at diagnosis		
20 or less	1 076 (57.1%)	1 665 (65.4%)
Above 20	645 (34.2%)	640 (25.2%)
Unknown	163 (8.7%)	239 (9.4%)
Choice of primary treatment		
Active surveillance	142 (7.5%)	248 (9.7%)
Radical prostatectomy	148 (7.9%)	184 (7.2%)
Radical radiotherapy	205 (10.9%)	333 (13.1%)
LHRH	986 (52.3%)	1434 (56.4%)
Other	403 (21.4%)	345 (13.6%)
PSA relapse	723 (38.4%)	842 (33.1%)
EAU risk group		
Low-risk	288 (15.3%)	480 (18.9%)
Intermediate-risk	625 (33.2%)	1014 (39.9%)
High-risk	971 (51.5%)	1050 (41.3%)
Use of other medication		
Andiabetic drugs	220 (11.7%)	745 (29.3%)
Antihypertensive drugs	1 152 (61.1%)	2 173 (85.4%)
NSAIDs	1 545 (82.0%)	2 218 (87.2%)
Aspirin	133 (7.1%)	448 (17.6%)
Type of ADT (categories not mutually exclusive)		
GnRH agonist/antagonist	1 497 (79.5%)	2 096 (82.4%)
Antiandrogens	1 116 (59.2%)	1 390 (54.6%)
Orchiectomy	210 (11.1%)	184 (7.2%)
Radiation therapy		
None	1 009 (53.6%)	1 111 (43.7%)
Yes	875 (46.4%)	1 433 (56.3%)
Socioeconomic status		
Employed	234 (12.4%)	269 (10.6%)
Unemployed	64 (3.4%)	47 (1.8%)
Retired	1 562 (82.9%)	2 210 (86.9%)
Unknown	24 (1.3%)	18 (0.7%)
Marital status		
Single/divorced/widow	626 (33.2%)	618 (24.3%)
Married/registered partnership	1 258 (66.8%)	1 926 (75.7%)

### Risk of prostate cancer death by statin use before ADT initiation

Statin use before ADT initiation was not associated with prostate cancer-specific survival (HR 1.12; 0.95 CI 0.96–1.31) (Table 2). No dose-dependence by yearly dosing was observed, either.

**Table 2:** Risk of prostate cancer death by statin use before ADT in a cohort of prostate cancer patients treated with ADT.

		Risk of PCa death	
Statin use before ADT	No of participants/PCa deaths	Age-adjusted	Multivariable adjusted*
None	2 904/593	Reference	Reference
Any	1 524/241	0.97 (0.84–1.13)	1.12 (0.96–1.31)
Intensity of statin use			
First tertile (below 120 DDD/year)	508/82	0.90 (0.71–1.13)	1.09 (0.86–1.38)
Second tertile (120–200 DDD/year)	510/90	1.07 (0.86–1.34)	1.19 (0.95–1.50)
Third tertile (above 200 DDD/year)	506/69	0.94 (0.74–1.22)	1.07 (0.83–1.38)

*Calculated using Cox regression with adjustment for age, tumor risk group, randomization group, use of other medication (antidiabetic and antihypertensive drugs, NSAIDs), and whether participants received radiation therapy in addition to ADT.

### Risk of PSA relapse after ADT initiation by statin use

Statin use after ADT initiation was linked to a decreased risk of PSA relapse (HR 0.73; 0.95 CI 0.65–0.82) when adjusted by age, randomization group, medications, and PCa risk group. In lag-time analysis, risk decrease remained statistically significant in the 1-year lag-time analysis.

### Risk of prostate cancer death by statin use after ADT initiation

In age-adjusted and multivariable-adjusted analyses, statin use after ADT was associated with a decreased risk of prostate cancer death (HR 0.82; 0.95 CI 0.69–0.96). Median PCa survival times after ADT initiation were 6.8 and 5.9 years for statin users and non-users, respectively. The association was dose-dependent and was observed most clearly in high-intensity statin use (HR 0.58; 0.95 CI 0.44–0.76) for men who had used at least 210 DDD/year. No significant risk decrease was observed in the lowest intensity tertile (HR 0.94; 0.95 CI 0.69–1.29) for men who had used 92 DDD/year or less (Table 3). Similar decreasing risk trends were observed also by cumulative DDD amount and years of statin use (Supplementary Table 1).

**Table 3:** Risk of prostate cancer death and PSA relapse by statin use after ADT initiation in a cohort of prostate cancer patients treated with ADT.

		Risk of prostate cancer death			
	No of participants/deaths	Age-adjusted	Multivariable adjusted	1-year lag-time	3-year lag-time
Statin use after ADT		HR (95% CI)	HR (95% CI)*	HR (95% CI)*	HR (95% CI)*
None	2707/582	Reference	Reference	Reference	Reference
Any	1721/252	0.68 (0.59–0.80)	0.82 (0.69–0.96)	0.89 (0.76–1.04)	0.90 (0.77–1.06)
Intensity of statin use (DDDs/year)					
First tertile (below 92 DDD/year)	574/160	0.83 (0.61–1.14)	0.94 (0.69–1.29)	0.99 (0.71–1.37)	0.86 (0.58–1.29)
Second tertile (92–210 DDD/year)	572/70	0.60 (0.47–0.75)	0.67 (0.53–0.84)	0.73 (0.57–0.93)	0.87 (0.67–1.15)
Third tertile (above 210 DDD/year)	575/22	0.48 (0.37–0.63)	0.58 (0.44–0.76)	0.88 (0.69–1.14)	0.93 (0.68–1.26)
Risk of PSA relapse					
Statin use after ADT	No of participants/PSA relapses	Age-adjusted	Multivariable adjusted*	1-year lag-time	3-year lag-time
None	2707/957	Reference	Reference	Reference	Reference
Any	1721/608	0.65 (0.58–0.72)	0.73 (0.65–0.82)	0.85 (0.76–0.95)	0.97 (0.86–1.09)

*Calculated using Cox regression with adjustment for age, tumor risk group, randomization group, use of other medication (antidiabetic and antihypertensive drugs, NSAIDs), and whether participants received radiation therapy in addition to ADT.

In the lag-time analysis, the risk decrease was slightly attenuated and no statistically significant difference was observed (Table 3).

### Subgroup analyses

**Fig. 2:**
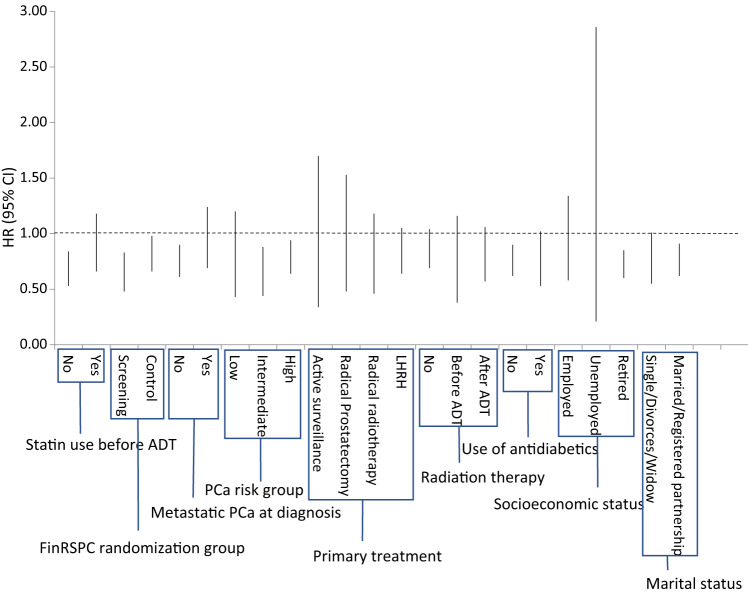
Risk of prostate cancer death by statin use after ADT stratified by subgroups. The vertical lines in figure represent 95% confidence intervals (CI).

No statistically significant effect modification was observed by FinRSPC study arm, PCa risk group, use of antidiabetic drugs, or use of radiation therapy in addition to ADT in stratified analyses (Table 4 and Fig. 2). Statin use after ADT initiation was associated with decreased mortality also in almost all subgroups.

**Table 4:** Risk of prostate cancer death by statin use after ADT stratified by various baseline variables.

		Participants/deaths	Risk of PCA death among ADT treated patients with statin use	
			Age-adjusted	Multivariable adjusted
			HR (95% CI)	HR (95% CI)*
FinRSPC randomization group	Control arm	2815/547	0.70 (0.58–0.84)	0.80 (0.66–0.98)
	Screening arm	1613/287	0.64 (0.49–0.84)	0.63 (0.48–0.83)
Statin use before ADT	No	2904/593	0.58 (0.46–0.73)	0.67 (0.53–0.84)
	Yes	1524/241	0.68 (0.51–0.91)	0.88 (0.66–1.18)
Metastatic PCa at diagnosis	No	3871/501	0.75 (0.62–0.90)	0.74 (0.61–0.90)
	Yes	556/333	0.88 (0.66–1.17)	0.93 (0.69–1.24)
PCa risk group**	Low risk	768/72	0.68 (0.41–1.11)	0.71 (0.43–1.20)
	Intermediate risk	1639/150	0.72 (0.51–1.01)	0.62 (0.44–0.88)
	High risk	2021/612	0.77 (0.64–0.92)	0.78 (0.64–0.94)
Choice of primary treatment	Active surveillance	390/31	0.76 (0.35–1.63)	0.76 (0.34–1.70)
	Radical prostatectomy	332/60	0.75 (0.42–1.31)	0.85 (0.48–1.53)
	Radical radiotherapy	538/90	0.74 (0.48–1.16)	0.74 (0.46–1.18)
Radiation therapy	No	2172/582	0.70 (0.58–0.86)	0.85 (0.69–1.04)
	Before ADT	299/62	0.70 (0.41–1.20)	0.67 (0.38–1.16)
	After ADT	1957/190	0.82 (0.61–1.11)	0.77 (0.57–1.06)
Use of antidiabetic drugs	No	3463/661	0.68 (0.57–0.82)	0.74 (0.62–0.90)
	Yes	965/173	0.68 (0.50–0.94)	0.74 (0.53–1.02)
Socioeconomic status	Employed	503/119	0.79 (0.53–1.18)	0.88 (0.58–1.34)
	Unemployed	111/23	0.53 (0.15–1.83)	0.77 (0.21–2.86)
	Retired	3772/677	0.66 (0.56–0.79)	0.71 (0.60–0.85)
Marital status	Single/divorced/widow	1244/263	0.67 (0.50–0.89)	0.74 (0.55–1.01)
	Married/registered partnership	3184/571	0.70 (0.59–0.84)	0.75 (0.62–0.91)

*Calculated using Cox regression with adjustment for age, tumor risk group, randomization group, use of other medication (antidiabetic and antihypertensive drugs, NSAIDs), and whether participants received radiation therapy in addition to ADT.

**Low risk: Gleason <7, T1/2 and PSA below 10, Intermediate risk: Gleason 7, T3 or PSA between 10 and 20, High risk: Gleason >7, T4, M+ or PSA above 20.

### Risk of death (all-cause mortality) by statin use before and after ADT initiation

Statin use after ADT initiation, but not before, was associated with decreased all-cause mortality (HR 0.84; 0.95 CI 0.76–0.93) (Table 5). The risk decrease was dose-dependent and was observed most clearly in high-intensity statin users.

**Table 5:** All-cause mortality by statin use after and before ADT in a cohort of prostate cancer patients treated with ADT.

	Statin use after ADT			Statin use before ADT		
	Participants/deaths	Age-adjusted	Multivariable adjusted	Participants/deaths	Age-adjusted	Multivariable adjusted
		HR (95% CI)	HR (95% CI)*		HR (95% CI)	HR (95% CI)*
None	2695/1226	Reference	Reference	2904/1347	Reference	Reference
Any	1733/701	0.79 (0.72–0.88)	0.84 (0.76–0.93)	1524/580	1.09 (0.99–1.20)	1.13 (1.02–1.25)
Amount of statin use (DDD)						
First tertile**	574/402	0.81 (0.66–1.00)	0.84 (0.68–1.04)	508/222	1.10 (0.95–1.26)	1.17 (1.02–1.36)
Second tertile	572/207	0.70 (0.61–0.80)	0.71 (0.62–0.82)	510/192	1.06 (0.91–1.24)	1.08 (0.93–1.26)
Third tertile	575/83	0.58 (0.49–0.68)	0.61 (0.51–0.71)	506/166	1.11 (0.94–1.30)	1.12 (0.95–1.32)

*Calculated using Cox regression with adjustment for age, tumor risk group, randomization group, use of other medication (antidiabetic and antihypertensive drugs, NSAIDs), and whether participants received radiation therapy in addition to ADT.

**Tertiles were defined as follows for statin use after/before ADT: first tertile below 92/120 DDD/year; Second tertile 92-210/120-200; third tertile above 210/200.

### Sensitivity analyses

To estimate whether risk estimates among statin users were affected by presumably increased cardiovascular mortality among statin users, we performed Fine and Gray competing risks regression analysis with death due to cardiovascular disease (ICD-10 codes I20–I25) as the competing outcome. In this analysis statin use before ADT was associated with a slightly elevated risk of PCa death (HR 1.17, 95% CI 0.99–1.38) whereas statin use after ADT continued to be associated with lowered risk (HR 0.68, 95% CI 0.58–0.80). Therefore lowered risk of PCa death is unlikely to be explained by a concomitantly elevated risk of cardiovascular death.

To assess how statin use at the time of ADT initiation may associate with PCa survival, we performed analysis including only statin use at baseline, i.e., the first year of ADT use, with follow-up starting at the second year of follow-up. In this analysis, statin use was not associated with PCa death (HR 1.02, 95% CI 0.85–1.24) confirming that statin users at baseline are at equal risk of dying of PCa compared to non-users, with no bias to favor statin users.

## Discussion

We have shown in a cohort of FinRSPC PCa patients that statin use after initiation of ADT, but not before, is associated with improved prostate cancer survival. The risk decrease remained even after adjusting for co-medications, PCa risk group, FinRSPC randomization group and additional radiation therapy and the risk reduction was dose-dependent. Especially, the finding that survival benefit was limited to statin use occurring after ADT supports synergism with ADT. This study clarifies our previous work observing increased prostate cancer survival, especially in ADT-treated patients using statins [10].

Recently, statin usage has been linked to a reduced risk of prostate cancer death, improved recurrence-free survival after radical treatment [1, 4, 5], decreased risk of advanced prostate cancer, and decreased risk of conversion of high-grade intraepithelial neoplasia to prostate cancer [12]. Also, a recent study published by Longo et al found fluvastatin to induce PCa cell death in vitro [13]. There are few studies assessing statins’ effect on ADT-treated patients [14, 15]. In these previous studies, the main results have been in line with our present study with HRs for PCa-specific mortality ranging from 0.64 to 0.76. In this study, we were able to assess the timing of statin use in relation to the initiation of ADT. Also, statins have been associated with decreased risk of clinically significant PCa, but not overall PCa risk [16]. However, there have also been contrary results, thus uncertainty remains [17]. Considering the previous studies, we hypothesized that the beneficial effects of statins relate to cancer progression, which would explain the difference in survival. In three previous studies, statin use has been linked to better survival among ADT-treated patients [10, 18, 19]. These studies suggest that statins may enhance the therapeutic effect of ADT. In contrast, one recent Danish study [20] failed to find any differences in progression-free survival or risk of progression between statin users and non-users in PCa patients primarily treated with ADT.

Prostate cancer is known to be dependent on androgens and ADT is commonly used to manage advanced prostate cancer. Cholesterol, instead, is a precursor for androgen synthesis, thus it would be logical to assume that cholesterol-lowering statins target androgen synthesis. Harshman et al. proved that statins compete with androgens for influx by SLCO2B1 transporter thus inhibiting tumor’s androgen supply [21]. In vitro studies have also found statins to enhance the effects of androgen-targeted drugs abiraterone acetate and enzalutamide [22]. These findings suggest that statins may improve the treatment outcomes of prostate cancer patients managed with ADT.

Epidemiologic studies assessing the association between statins and cancer survival have many potential sources of bias. Statin users may differ by socioeconomic status and health behavior from non-users. In this study, we were able to adjust for established risk factors for PCa death (TNM stage, Gleason score, PSA at diagnosis, other medications). Still, residual confounding may occur. Nevertheless, residual confounding would be expected to affect statin use similarly regardless of timing in relation to ADT. Therefore, our finding that only statin usage after ADT, not before it, was associated with improved PCa survival in a multivariable-adjusted model, supports a need for further studies to assess possible causality.

To date, only one study has assessed the effect of statins on prostate cancer survival in a randomized setting in prostate cancer patients [23]. Atorvastatin did not significantly reduce PSA or Ki-67 (a marker of cellular proliferation) overall compared to placebo, but a significant decreasing trend in tumor proliferation marker Ki-67 by the length of atorvastatin exposure was observed. The study population consisted of men scheduled for radical prostatectomy and atorvastatin intervention was used only before surgery for a median of 21 days. Thus, the study setting differs from our study population consisting only of ADT-treated prostate cancer patients. Our current study suggests that the effect of statins on PCa may be greater in the context of ADT.

We were able to analyze statins’ effect in our large population-based cohort consisting of 4428 men starting ADT. Detailed information on medication use and timing of purchases allowed us to analyze separately statin use before and after the beginning of ADT. Statins cannot be purchased over the counter in Finland. Thus, the information on statin purchases is comprehensively registered by the prescription database. Also, clinical characteristics of prostate cancer cases and causes of death were obtained from comprehensive and reliable national databases and supplemented with patient files.

We were not able to analyze the effects of physical activity, diet, and use of health services reliably as the data was missing. These unmeasured variables may have caused residual confounding. In the future, randomized clinical trials are needed to definitely evaluate the causal impact of statins on PCa survival. Further, statins’ efficacy against prostate cancer may depend on the tumor’s genotype, as certain PCa subtypes have been linked to increased lipid and cholesterol production [24]. We did not have information on PCa genotypes, thus we were unable to assess statins by genotype.

## Conclusion

In a cohort of FinRSPC prostate cancer patients, statin use after initiation of ADT, but not before it, was associated with improved prostate cancer prognosis. This finding is in line with previous in vitro studies reporting possible synergy between statins and ADT. Randomized trials are needed to confirm or refute the survival benefit of statins among ADT-treated PCa patients.

## Supplementary information

Supplementary Table 1
