# Supplementary material for: Prostate cancer prognosis after initiation of androgen deprivation therapy among statin users. A population-based cohort study
Source: Prostate Cancer Prostatic Dis. 2021 Mar 31;24(3):917–24. doi: 10.1038/s41391-021-00351-2 (PMC8384625; doi:10.1038/s41391-021-00351-2)
Supplement: Supplementary file 1 — Supplementary Table 1 [file 41391_2021_351_MOESM1_ESM.docx]

Supplementary Table

Supplementary table 1. Risk of prostate cancer death by amount and duration of statin use after initiation of androgen-deprivation therapy. Cohort of 8 253 prostate cancer cases from the Finnish Randomized Study of Screening for Prostate Cancer.

|  |  | Age-adjusted | Multivariable  adjusted* |
| --- | --- | --- | --- |
| Amount of statin use (DDD) | N of participants/deaths |  |  |
| 1st tertile (below 820 DDD) | 573/142 | 0.72 (0.60-0.86) | 0.78 (0.65-0.94) |
| 2nd tertile (820-2156 DDD) | 573/82 | 0.67 (0.52-0.85) | 0.72 (0.56-0.93) |
| 3rd tertile (above 2156 DDD) | 575/28 | 0.55 (0.37-0.81) | 0.59 (0.39-0.88) |
| Statin use (years) |  |  |  |
| 1st tertile (below 9 years) | 505/54 | 0.77 (0.63-0.94) | 0.80 (0.65-0.99) |
| 2nd tertile (9-11 years) | 525/66 | 0.64 (0.25-1.64) | 0.70 (0.27-1.79) |
| 3rd tertile (above 12 years) | 691/132 | 0.39 (0.05-2.90) | 0.40 (0.05-3.02) |

* Calculated using Cox regression with adjustment for age, tumor risk group, randomization group, use of other medication (antidiabetic and antihypertensive drugs, NSAIDs) and whether participants received radiation therapy in addition to ADT
